# Biological Interpretable Machine Learning Model for Predicting Pathological Grading in Clear Cell Renal Cell Carcinoma Based on CT Urography Peritumoral Radiomics Features

**DOI:** 10.3390/bioengineering12101125

**Published:** 2025-10-20

**Authors:** Dingzhong Yang, Haonan Mei, Panpan Jiao, Qingyuan Zheng

**Affiliations:** 1 Experimental Teaching and Engineering Training Center, South-Central Minzu University, Wuhan 430074, China; 2 The First Clinical College, Wuhan University, Wuhan 430060, China

**Keywords:** clear cell renal cell carcinoma, ISUP grading, peritumoral area, radiomics, machine learning

## Abstract

**Background:** The purpose of this study was to investigate the value of machine learning models for preoperative non-invasive prediction of International Society of Urological Pathology (ISUP) grading in clear cell renal cell carcinoma (ccRCC) based on CT urography (CTU)-related peritumoral area (PAT) radiomics features. **Methods:** We retrospectively analysed 328 ccRCC patients from our institution, along with an external validation cohort of 175 patients from The Cancer Genome Atlas. A total of 1218 radiomics features were extracted from contrast-enhanced CT images, with LASSO regression used to select the most predictive features. We employed four machine learning models, namely, Logistic Regression (LR), Multilayer Perceptron (MLP), Support Vector Machine (SVM), and Extreme Gradient Boosting (XGBoost), for training and evaluation using Receiver Operating Characteristic (ROC) analysis. The model performance was assessed in training, internal validation, and external validation sets. **Results:** The XGBoost model demonstrated consistently superior discriminative ability across all datasets, achieving AUCs of 0.95 (95% CI: 0.92–0.98) in the training set, 0.93 (95% CI: 0.89–0.96) in the internal validation set, and 0.92 (95% CI: 0.87–0.95) in the external validation set. The model significantly outperformed LR, MLP, and SVM (*p* < 0.001) and demonstrated prognostic value (Log-rank *p* = 0.018). Transcriptomic analysis of model-stratified groups revealed distinct biological signatures, with high-grade predictions showing significant enrichment in metabolic pathways (DPEP3/THRSP) and immune-related processes (lymphocyte-mediated immunity, MHC complex activity). These findings suggest that peritumoral imaging characteristics provide valuable biological insights into tumor aggressiveness. **Conclusions:** The machine learning models based on PAT radiomics features of CTU demonstrated significant value in the non-invasive preoperative prediction of ISUP grading for ccRCC, and the XGBoost modeling had the best predictive ability. This non-invasive approach may enhance preoperative risk stratification and guide clinical decision-making, reducing reliance on invasive biopsy procedures.

## 1. Introduction

Renal cell carcinoma (RCC), a malignant neoplasm arising from renal tubular epithelium, represents a significant global health burden with approximately 431,000 new cases reported worldwide in 2020, demonstrating a notable male predominance [1,2]. Among RCC subtypes, clear cell RCC (ccRCC) accounts for 70% of cases and exhibits particularly aggressive clinical behavior [3]. The WHO/International Society of Urological Pathology (ISUP) grading system, established in 2016, serves as the current gold standard for ccRCC prognostication [4]. Grades I to III are determined by evaluating nucleolar prominence, while the presence of highly atypical pleomorphic cells and/or sarcomatoid or rhabdoid morphology indicates Grade IV. Low-grade (ISUP I-II) tumors are associated with a favorable prognosis, whereas high-grade (ISUP III-IV) tumors correlate with poorer outcomes and require more aggressive treatment [5]. Therefore, tumor grading plays a crucial role in guiding therapeutic decisions. Preoperative pathological grading assessment typically relies on renal biopsy. However, due to the high heterogeneity of ccRCC, biopsy results may only represent a portion of the lesion and fail to fully reflect the nuclear grade of the entire tumor. Additionally, this invasive procedure carries risks such as bleeding, low reproducibility, and potential complications [6,7]. Therefore, developing a non-invasive and accurate histological grading assessment method for ccRCC has significant clinical significance and application value [8].

Radiomics holds great promise in tumor staging, grading, and molecular characterization [9,10,11]. Many radiomics studies have focused on predicting nuclear grading to assess tumor aggressiveness [12,13,14]. In a previous study, we proposed a non-invasive approach using radiomic features from a large cohort to determine ISUP grading [15]. We collected CT urography (CTU) images from 507 ccRCC patients across two cohorts, achieving an AUC of 0.91 in the validation set, outperforming other studies. For example, Cui et al. reported an accuracy of less than 80% in predicting pathological grade using multiparametric MRI and multiphase CT data [16]. Zheng et al. developed a model with 20 radiomic features in a single cohort, achieving an AUC of 0.846 in the validation set [17]. However, these models relied solely on intratumoral imaging features and did not explore the value of peritumoral area (PAT) radiomics. Li et al. evaluated radiomic features from both intratumoral and peritumoral regions and achieved an AUC of 0.80 in the training set, indicating limited but promising discriminative ability of PAT features [18]. Currently, PAT radiomics is increasingly being applied in the diagnosis, grading, and prognosis of other cancers, highlighting its potential value [19,20,21].

This study evaluates the predictive value of PAT radiomics features for ISUP grading in ccRCC patients across two independent cohorts. We developed a non-invasive radiomics-based approach to determine ISUP grading, which could potentially improve clinical decision-making, surgical planning, and patient prognosis.

## 2. Materials and Methods

### 2.1. Study Population

**Figure 1:**
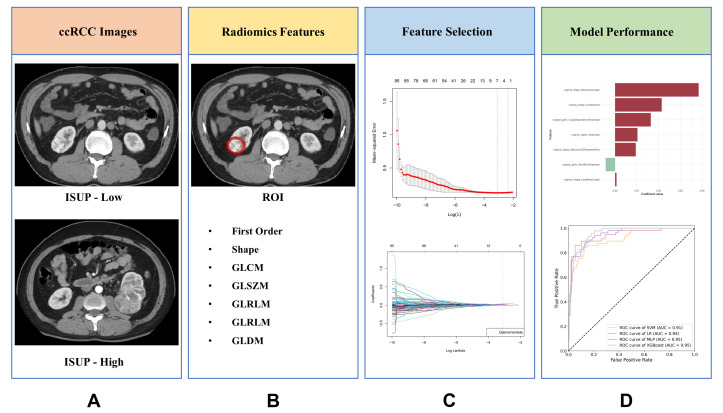
The key methodological steps of the study. (**A**) Acquisition of ccRCC CT imaging data; (**B**) tumor segmentation and radiomic feature extraction from defined regions of interest (ROIs), the red circle indicates the ROI on the tumor area; (**C**) dimensionality reduction and feature selection through statistical and machine learning approaches; (**D**) predictive model development and validation across multiple cohorts.

**Figure 2:**
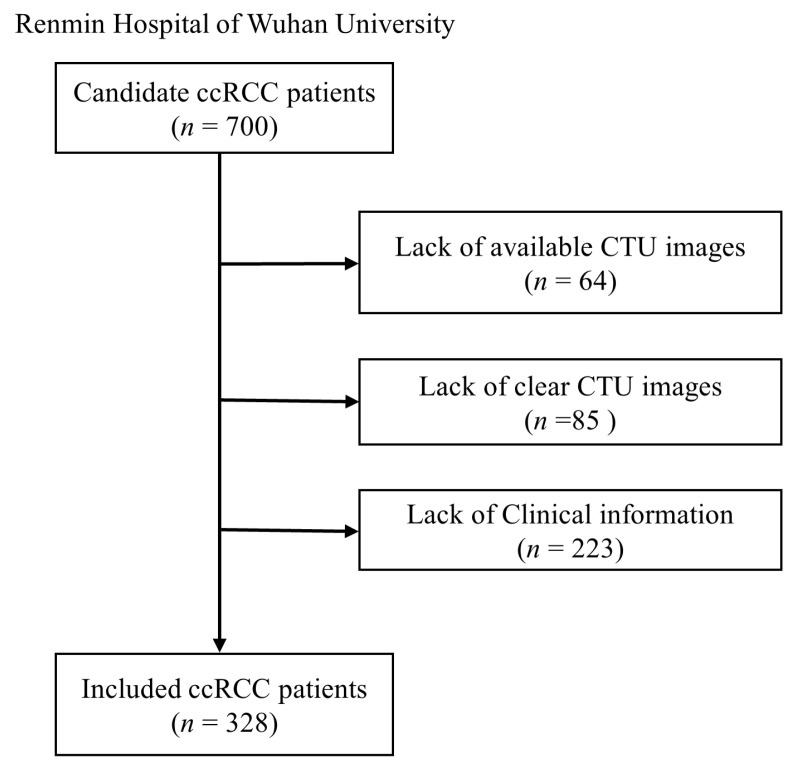
Recruitment process of RHWU cohort.

The study design is schematically presented in Figure 1. Conducted in accordance with the Declaration of Helsinki, this study received ethical approval from Renmin Hospital of Wuhan University (WDRY2022-K077). The Clinical Research Ethics Committee, RHWU waived the need for Informed Consent due to the retrospective nature of the study. We retrospectively analyzed consecutive ccRCC patients who underwent surgical resection between 2020–2024, with pathological confirmation. After excluding ineligible cases (85 for poor image quality, 64 for missing CTU images, and 223 for incomplete records), 328 patients comprised the primary cohort. The TCGA cohort provided 175 additional cases for external validation (obtained from The Cancer Imaging Archive (TCIA, https://www.cancerimagingarchive.net/, accessed on 10 April 2025)). Inclusion criteria required: (i) pathologically confirmed ccRCC with ISUP grade, (ii) no prior systemic therapy, (iii) no concurrent malignancies, and (iv) complete demographic/staging data. Figure 2 illustrates the selection process.

### 2.2. CTU Images Collection

Thin-section CTU images were acquired at 0.625 mm slice thickness from all enrolled patients with pathologically confirmed ccRCC. Two board-certified abdominal radiologists (each with 7 years of subspecialty experience) independently evaluated all CTU images in a blinded fashion, without access to clinical or pathological data. To ensure consensus in interpretation, any discordant findings were resolved through review by a senior radiologist with 12 years of genitourinary imaging expertise. All images were archived in standardized DICOM format for subsequent analysis.

### 2.3. ISUP Evaluation

The ISUP grading for all enrolled patients was determined through comprehensive evaluation of pathological diagnosis reports. In accordance with established clinical standards, we classified ISUP grades I–II as low-grade and grades III–IV as high-grade tumors [22]. For cases exhibiting heterogeneous tumor grading (multiple grades present within the same specimen), the highest observed grade was documented as the representative grade for analytical purposes, ensuring conservative clinical correlation with imaging findings.

### 2.4. PAT Annotation

All clear cell renal cell carcinoma lesions were manually segmented using ITK-SNAP software (version 3.8.0) with slice-by-slice delineation of tumor boundaries [23]. Two fellowship-trained abdominal radiologists independently performed regions of interest (ROI) delineation on corticomedullary phase images, with all segmentations subsequently reviewed and corrected as needed by a senior radiologist. The intratumoral region encompassed the entire tumor volume, including necrotic, cystic, and hemorrhagic components, while carefully excluding adjacent normal renal parenchyma, perirenal fat, and sinus fat. Following established protocols, we defined the PAT as a uniform 5 mm expansion beyond the tumor boundary, capturing potentially informative transitional tissue16. All imaging data were converted from DICOM to NIfTI (.nii) format for subsequent radiomic analysis.

### 2.5. Radiomics Feature Extraction

Radiomic feature extraction was performed using PyRadiomics (version 3.1.0) implemented in Python (version 3.10.11) [24]. From each segmented region, we systematically extracted seven comprehensive feature classes: (1) first-order statistical features quantifying intensity distributions; (2) 2D shape descriptors characterizing planar morphology; (3) 3D volumetric shape features; (4) gray-level co-occurrence matrix (GLCM) features assessing spatial relationships; (5) gray-level size zone matrix (GLSZM) features evaluating connected voxel patterns; (6) gray-level run length matrix (GLRLM) features measuring texture granularity; and (7) gray-level dependence matrix (GLDM) features analyzing gray-level dependencies. All extracted features were systematically cataloged and stored in standardized CSV format for subsequent machine learning analysis.

### 2.6. Feature Dimension Reduction

All extracted radiomic features underwent standardization using the StandardScaler function to ensure consistent scaling. We assessed feature reliability through inter- and intra-observer intraclass correlation coefficients (ICCs), retaining only features demonstrating excellent reproducibility (ICC > 0.75). Subsequent univariate analysis employed Student’s *t*-tests for normally distributed features and Mann–Whitney U tests for non-parametric distributions, with statistically significant features (*p* < 0.05) selected for further analysis. To address dimensionality, we implemented least absolute shrinkage and selection operator (LASSO) regression with 10-fold cross-validation. The optimal regularization parameter (λ) was determined by identifying the value yielding minimal cross-validation error. Features with non-zero coefficients after LASSO regularization were incorporated into final predictive model construction and subsequent analyses.

### 2.7. Machine Learning Models Development

The RHWU cohort was randomly stratified into training (70%) and internal validation (30%) sets, while maintaining the TCGA cohort as an independent external validation set. Four distinct machine learning models were developed to predict ISUP grading from corticomedullary phase CTU images: (1) logistic regression (LR) as a baseline linear model; (2) support vector machine (SVM) with radial basis function kernel; (3) multilayer perceptron (MLP) neural network; and (4) extreme gradient boosting (XGBoost) ensemble method. Model performance was rigorously evaluated using receiver operating characteristic (ROC) analysis, with area under the curve (AUC) serving as the primary metric for diagnostic accuracy assessment.

### 2.8. Biological Interpretability Analysis

To validate the biological significance of our model, we integrated RNA sequencing data from 175 ccRCC patients in the TCGA database. Differential expression analysis (DESeq2, FDR < 0.05) was performed between model-predicted high- and low-grade groups, identifying significantly differentially expressed genes. Gene Ontology (GO) enrichment analysis was conducted using clusterProfiler. All analyses were performed using R 4.2.0, with visualizations generated Via ggplot2.

### 2.9. Statistical Analysis

All statistical analyses were conducted using Python 3.1.0 with the following scientific computing packages: scikit-learn (v1.2.2) for machine learning implementation, SciPy (v1.10.1) for fundamental statistical computations, and statsmodels (v0.13.5) for advanced statistical modeling. We employed two-tailed hypothesis testing throughout the study, with a threshold of *p* < 0.05 establishing statistical significance.

## 3. Results

### 3.1. Patient Characteristics

The demographic, clinical, and pathological characteristics of the study cohorts are presented in Table 1. The baseline data include comprehensive parameters for the RHWU training, internal validation (*n* = 328), and external TCGA validation (*n* = 175) cohorts, demonstrating balanced distribution of key prognostic factors across all groups.

### 3.2. PAT Radiomics Feature Extraction

Our comprehensive radiomic analysis using PyRadiomics yielded 1218 quantitative imaging features across seven distinct categories (first-order statistics, shape features, and texture matrices). The complete inventory of extracted features, including their mathematical definitions and extraction parameters, has been systematically documented in Supplementary Table S1.

### 3.3. PAT Feature Selection

**Figure 3:**
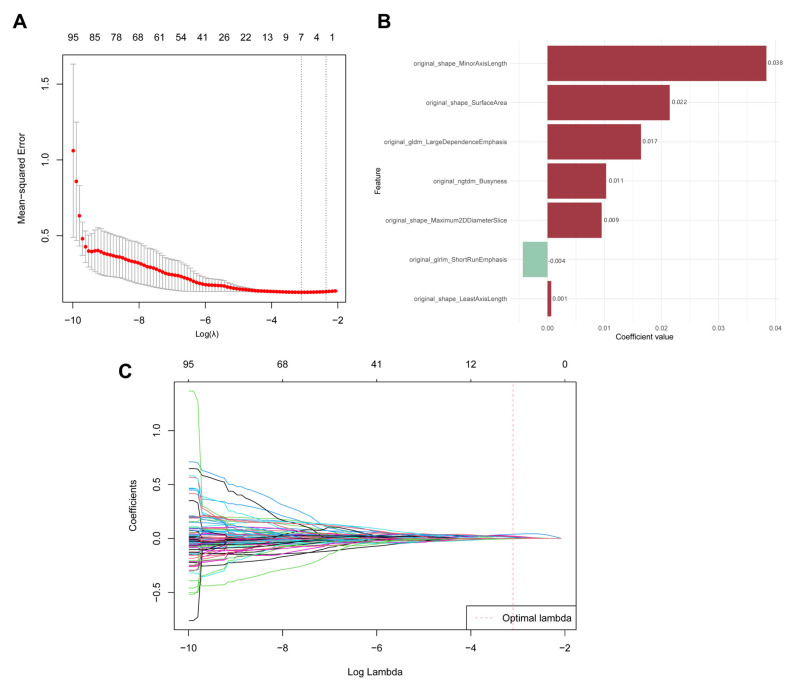
LASSO regression results of PAT radiomics features. (**A**) Coefficient path plot; (**B**) characteristic coefficient map; (**C**) LASSO regression cross-validation curve.

From the initial set of 1218 radiomics features, LASSO regression with 10-fold cross-validation identified seven robust predictors significantly associated with ISUP grading (Figure 3). The selected features coefficients and importance weights detailed in Table 2. These PAT-derived features particularly highlighted original_shape_MinorAxisLength, original_shape_SurfaceArea and original_gldm_LargeDependenceEmphasis, suggesting their potential as imaging biomarkers for ccRCC aggressiveness.

### 3.4. Performance of Machine Learning Models

**Figure 4:**
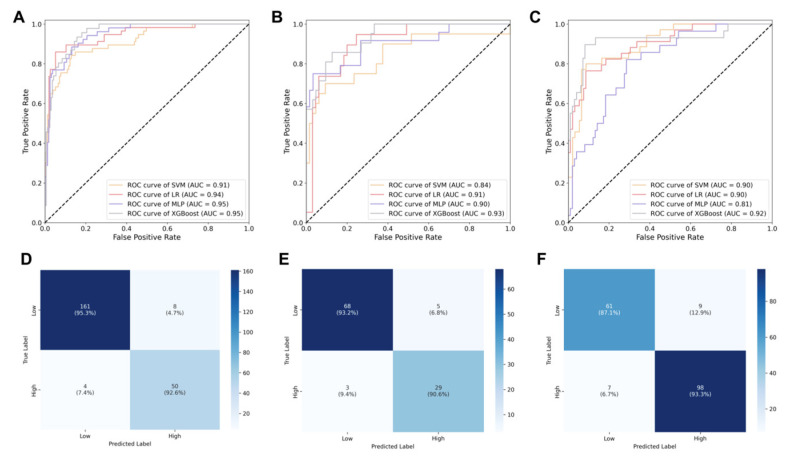
Performance evaluation of machine learning models for ISUP grade prediction. ROC curves demonstrate the predictive performance of LR, MLP, SVM, and XGBoost models in (**A**) training, (**B**) internal and (**C**) external validation cohorts. Corresponding confusion matrices showing classification accuracy are presented for (**D**) training, (**E**) internal and (**F**) external validation sets.

The machine learning models demonstrated robust performance in predicting ISUP grading for ccRCC patients. Baseline clinical characteristics showed no significant differences between training and test sets (all *p* > 0.05, Table 3). In the training cohort, all models achieved strong predictive accuracy, with AUCs ranging from 0.91 (SVM, 95% CI: 0.88–0.94) to 0.95 (XGBoost and MLP, 95% CI: 0.92–0.98 and 0.91–0.97, respectively). The performance remained consistent in the internal validation, where XGBoost maintained the highest AUC (0.93, 95% CI: 0.89–0.96), followed by LR (0.91, 95% CI: 0.88–0.94) and MLP (0.90, 95% CI: 0.87–0.92), while SVM showed slightly reduced performance (0.84, 95% CI: 0.81–0.87). External validation with the TCGA cohort confirmed the generalizability of results, with XGBoost demonstrating superior performance (AUC = 0.92, 95% CI: 0.87–0.95), closely followed by LR and SVM (both AUC = 0.90), while MLP showed more variability (AUC = 0.81). The complete performance characteristics are illustrated in Figure 4A–C.

The XGBoost model demonstrated superior predictive accuracy across all datasets, with classification accuracies of 0.95 (95% CI: 0.88–0.94) in training set, 0.92 (95% CI: 0.89–0.96) in testing set, and 0.91 (95% CI: 0.87–0.94) in validation set. DeLong’s test for ROC curve comparison confirmed XGBoost’s statistically significant outperformance over LR, SVM, and MLP models (all *p* < 0.001). Complete performance metrics for all models are detailed in Table 4, while Figure 4D–F visually represents the classification outcomes through confusion matrices for each dataset.

**Figure 5:**
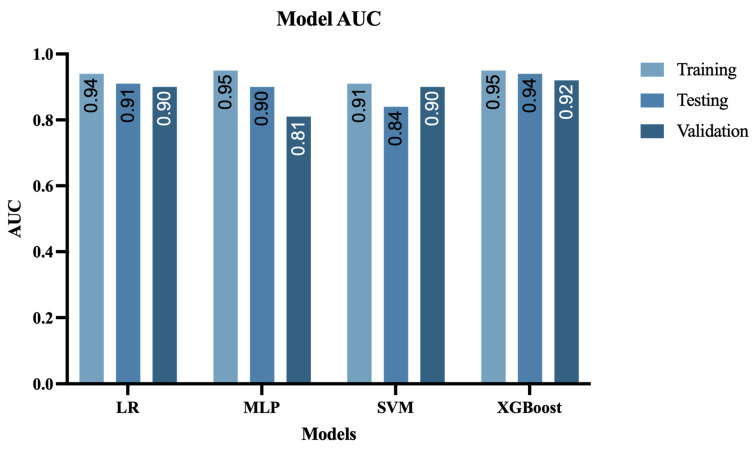
Comparative predictive performance of four machine learning models.

For the test set, we generated a forest plot of data categories, which clearly demonstrated XGBoost’s superior performance. Compared to other models, XGBoost showed higher mean AUC values with narrower confidence intervals, indicating more stable performance on test set data (Figure 5).

**Figure 6:**
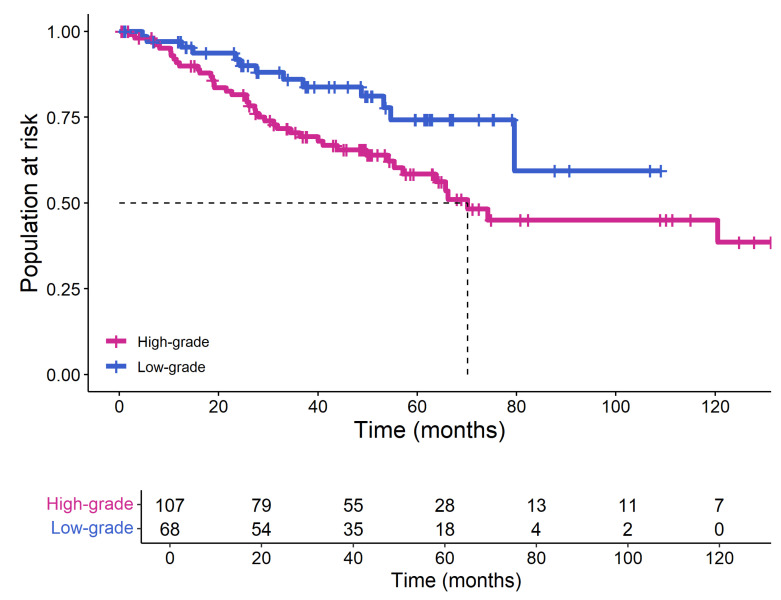
The prognostic value of pathological grades predicted by the XGBoost model. The figure below shows the number of high-grade and low-grade patients predicted by the XGBoost model. The dashed line indicates the time when 50% of the high-grade patient population is still at risk.

It is noteworthy that the pathological grade predicted by the XGBoost model still holds prognostic value, with the Kaplan–Meier curve demonstrating the ability to stratify ccRCC patients into high- and low-risk groups (Log-rank *p* = 0.018; Figure 6).

### 3.5. The Biological Interpretability of the Model

**Figure 7:**
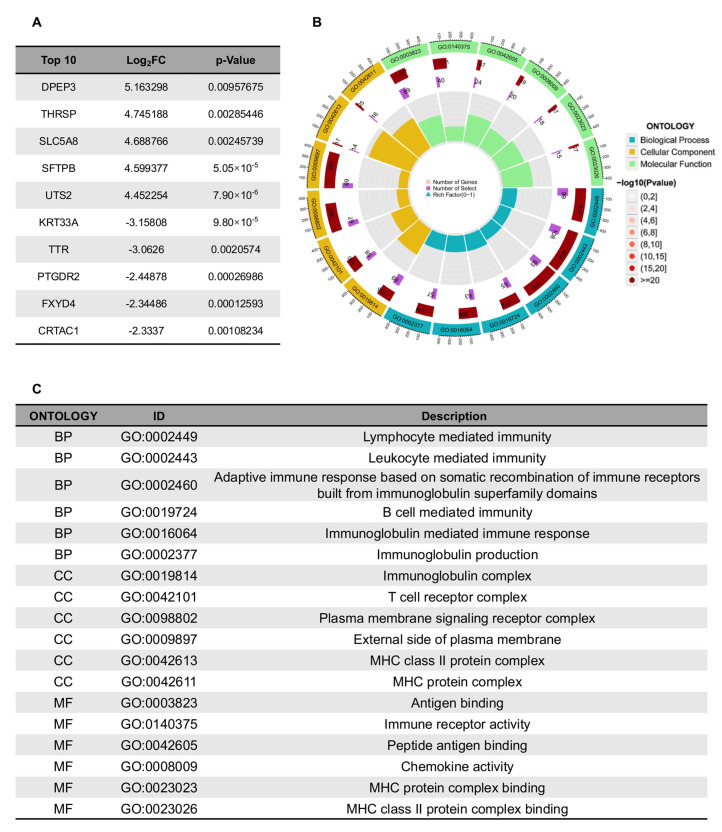
The biological interpretability of the machine learning model. (**A**) Top 10 most significant differential expressed genes. (**B**,**C**) Gene Ontology enrichment analysis.

To investigate the biological mechanisms underlying the model’s classification of high- and low-grade ccRCC, we performed differential gene expression analysis using RNA-seq data from TCGA. The top differentially expressed genes (DEGs) revealed distinct molecular signatures between the two groups (Figure 7A). Notably, DPEP3 (dipeptidase 3), THRSP (thyroid hormone responsive), and SLC5A8 (solute carrier family 5 member 8) were significantly upregulated in high-grade tumors, suggesting roles in tumor progression and metabolic reprogramming. Conversely, KRT33A (keratin 33A), TTR (transthyretin), and CRTAC1 (cartilage acidic protein 1) were downregulated, potentially reflecting loss of differentiation markers in aggressive disease.

GO enrichment analysis further demonstrated strong associations with immune-related processes (Figure 7B,C). The most enriched biological processes included lymphocyte-mediated immunity, adaptive immune response, and immunoglobulin production, indicating active immune modulation in the tumor microenvironment. Cellular component terms highlighted immunoglobulin complexes, MHC class II protein complexes, and T cell receptor complexes, reinforcing the involvement of antigen presentation and immune recognition [25]. Molecular function analysis revealed enrichment for antigen binding, immune receptor activity, and chemokine activity, suggesting that high-grade ccRCC may exhibit altered immune surveillance.

These findings align with emerging evidence that ccRCC aggressiveness is closely linked to immune evasion and metabolic dysregulation. The model’s ability to stratify tumors based on radiomic features may thus reflect underlying biological differences in immune infiltration and metabolic pathways, providing a plausible mechanistic basis for its predictive performance. Further studies integrating radiomics with multi-omics data could help elucidate these relationships in greater depth.

## 4. Discussion

In this study, we developed and validated machine learning models using PAT-related radiomics features to preoperatively predict ISUP grading in ccRCC patients. Our results demonstrated that PAT-derived radiomics features exhibited strong predictive performance, with XGBoost emerging as the optimal model (AUC: 0.95 in training, 0.93 in internal validation, and 0.92 in external validation sets). These findings suggested that PAT radiomics analysis could serve as a valuable non-invasive tool for tumor grading, potentially complementing current diagnostic approaches.

Several aspects of our findings warrant further discussion. First, the superior performance of PAT-based features aligns with growing evidence that tumor microenvironment characteristics significantly influence ccRCC aggressiveness [26,27,28]. Our LASSO-selected features, particularly texture-based parameters, may reflect underlying pathological changes in the tumor periphery that correlate with nuclear grade. This observation supports the biological plausibility of our approach, as previous studies have shown that peritumoral regions often harbor early signs of tumor invasion and microenvironment remodeling [29,30].

The outstanding performance of XGBoost compared to other models can be attributed to its ability to handle complex feature interactions and nonlinear relationships, which are characteristic of radiomics data. Our results corroborate recent findings, who similarly reported XGBoost’s superiority in radiomics-based cancer grading tasks [31,32,33]. The model’s consistent performance across both internal and external validation sets suggests robust generalizability, though the slight performance drop in external validation underscores the importance of multicenter validation studies.

Notably, our PAT-based model achieved higher accuracy (AUC: 0.92–0.95) compared to previous studies focusing solely on intratumoral features (typically AUC: 0.80–0.88) [14,15]. This improvement likely stems from the additional biological information captured in peritumoral regions, including early stromal changes and microvascular alterations that may precede detectable intratumoral changes. Our findings thus add to the growing body of evidence supporting the clinical value of peritumoral imaging analysis.

The integration of radiomics with transcriptomic data in our study provides compelling biological insights into the molecular underpinnings of our model’s predictive performance. The observed upregulation of DPEP3 and THRSP in high-grade tumors aligns with their established roles in tumor progression and metabolic reprogramming, while the downregulation of differentiation markers like KRT33A suggests a loss of epithelial characteristics in aggressive disease. Notably, the significant enrichment of immune-related pathways, particularly those involving antigen presentation and lymphocyte-mediated immunity, supports growing evidence that the peritumoral microenvironment plays a crucial role in ccRCC progression. These findings suggest that our PAT-based radiomics model may be capturing subtle imaging manifestations of these underlying biological processes, particularly the immune landscape that has been increasingly recognized as a key determinant of tumor behavior. The convergence of radiomic and transcriptomic signatures reinforces the biological plausibility of our approach and highlights the potential of radiomics to serve as a non-invasive proxy for molecular characterization. Future studies incorporating spatial transcriptomics or multiplex immunohistochemistry could further elucidate the precise relationships between imaging features and their cellular and molecular correlates in the peritumoral region.

Several limitations should be acknowledged. First, while our multicenter design strengthens the findings, the retrospective nature introduces potential selection biases. Second, manual ROI segmentation, though performed by experienced radiologists, may introduce variability; future studies could explore automated segmentation methods. Third, the biological mechanisms linking specific radiomics features to tumor grade remain to be fully elucidated through histopathological correlation studies.

Despite these limitations, our study has important clinical implications. The ability to accurately predict ISUP grade noninvasively could significantly impact clinical decision-making, particularly in cases where biopsy is contraindicated or yields inconclusive results. Furthermore, our PAT-based approach may help identify patients who would benefit from more aggressive surveillance or adjuvant therapies, potentially improving outcomes.

Future research directions should include: (1) prospective validation in larger, multicenter cohorts; (2) integration of PAT radiomics with other biomarkers (e.g., genomic data) to develop more comprehensive predictive models; and (3) investigation of the biological underpinnings of predictive radiomics features through correlative histopathological studies.

## 5. Conclusions

Our study demonstrates that PAT radiomics analysis using machine learning, particularly XGBoost, can effectively predict ISUP grading in ccRCC patients. These findings support the potential clinical utility of PAT-based radiomics as a noninvasive tool for tumor characterization, with possible applications in personalized treatment planning and patient stratification.

## Figures and Tables

**Table 1:** Demographic, clinical, and pathological characteristics of ccRCC patients in the RHWU and TCGA cohort.

Characteristics	RHWU (*N* = 328)	TCGA (*N =* 175)
**Age (years)**	59 (25, 84)	59 (26, 88)
**Gender**		
** Female**	124 (37.8%)	64 (36.5%)
** Male**	204 (62.2%)	111 (63.5%)
**pT stage**		
** pT1**	224 (68.3%)	92 (52.5%)
** pT2**	40 (12.1%)	19 (10.9%)
** pT3**	51 (15.6%)	61 (34.9%)
** pT4**	13 (4.0%)	3 (1.7%)
**pN stage**		
** pN0**	326 (99.4%)	74 (42.3%)
** pN1**	2 (0.6%)	3 (1.7%)
** pNx**	0 (0%)	98 (56.0%)
**pM stage**		
** pM0**	326 (99.4%)	144 (82.3%)
** pM1**	2 (0.6%)	25 (14.3%)
** pMx**	0 (0%)	6 (3.4%)
**pTNM stage**		
** Stage I**	225 (68.6%)	89 (50.9%)
** Stage II**	39 (11.9%)	15 (8.6%)
** Stage III**	51 (15.5%)	44 (25.1%)
** Stage IV**	13 (4.0%)	27 (15.4%)
**ISUP**		
** Low**	242 (73.8%)	71 (40.1%)
** High**	86 (26.2%)	104 (59.9%)

**Table 2:** Radiomics features associated with ISUP grading selected by LASSO regression.

Signature	Coefficients
original_shape_LeastAxisLength	0.000658846
original_shape_Maximum2DDiameterslice	0.009503959
original_shape_MinorAxisLength	0.038404090
original_shape_surfaceArea	0.021442057
original_gldm_LargeDependenceEmphasis	0.016409816
original_glrlm_shortRunEmphasis	−0.004333667
original_ngtdm_Busyness	0.010288175

**Table 3:** Comparison of clinical parameters between training set and testing set.

Variable	Overall, *N* = 328 ^1^	Training Set, *N* = 223 ^1^	Internal Set, *N* = 105 ^1^	*p*-Value ^2^
**Gender**				0.079
**Female**	124 (38%)	84 (38%)	40 (38%)	
**Male**	204 (62%)	139 (62%)	65 (52%)	
**Age**	59 (52, 66)	57 (51, 66)	61 (55, 66)	0.059
**pTNM stage**				0.096
**I**	225 (69%)	169 (76%)	56 (53%)	
**II**	39 (12%)	24 (11%)	15 (7%)	
**III**	51 (16%)	22 (10%)	29 (28%)	
**IV**	13 (4.0%)	8 (3%)	5 (12%)	
**ISUP Grade**				0.19
**Low-grade**	242 (74%)	169 (76%)	73 (70%)	
**High-grade**	86 (26%)	54 (24%)	32 (30%)	

^1^ Median (IQR) or frequency (%). ^2^ Pearson’s Chi-squared test; Wilcoxon rank sum test; Fisher’s exact test.

**Table 4:** Model performance summary.

Dataset	Model	AUC (95%CI)	Sensitivity (95%CI)	Accuracy (95%CI)
**Training**	LR	0.94 (0.90, 0.97)	0.92 (0.88, 0.94)	0.91 (0.88, 0.94)
	MLP	0.95 (0.91, 0.97)	0.93 (0.88, 0.95)	0.93 (0.89, 0.96)
	SVM	0.91 (0.88, 0.94)	0.90 (0.86, 0.94)	0.91 (0.88, 0.94)
	XGBoost	0.95 (0.92, 0.98)	0.95 (0.92, 0.97)	0.95 (0.88, 0.94)
**Testing**	LR	0.91 (0.88, 0.94)	0.90 (0.87, 0.93)	0.89 (0.86, 0.93)
	MLP	0.90 (0.87, 0.92)	0.88 (0.85, 0.91)	0.87 (0.85, 0.89)
	SVM	0.84 (0.81, 0.87)	0.83 (0.81, 0.87)	0.83 (0.81, 0.87)
	XGBoost	0.93 (0.89, 0.96)	0.93 (0.90, 0.95)	0.92 (0.89, 0.96)
**Validation**	LR	0.90 (0.86, 0.93)	0.85 (0.88, 0.94)	0.88 (0.86, 0.90)
	MLP	0.81 (0.79, 0.84)	0.80 (0.78, 0.84)	0.81 (0.78, 0.86)
	SVM	0.90 (0.88, 0.93)	0.86 (0.83, 0.89)	0.85 (0.82, 0.88)
	XGBoost	0.92 (0.87, 0.95)	0.87 (0.85, 0.90)	0.91 (0.87, 0.94)

## Data Availability

The Pyradiomics package is available for free on GitHub (https://github.com/AIM-Harvard/pyradiomics/ accessed on 15 May 2025). For ethical reasons, the data used in this study should be provided upon request to the corresponding author.

## Supplementary Materials

The following supporting information can be downloaded from https://www.mdpi.com/article/10.3390/bioengineering12101125/s1.

## Abbreviations

The following abbreviations are used in this manuscript:

RCC	Renal Cell Carcinoma
ccRCC	Clear Cell Renal Cell Carcinoma
ISUP	International Society of Urological Pathology
WHO	World Health Organization
PAT	Peritumoral Area
CTU	Computed Tomography Urography
DICOM	Digital Imaging and Communications in Medicine
ROI	Region of Interest
GLCM	Gray-Level Co-occurrence Matrix
GLSZM	Gray-Level Size Zone Matrix
GLRLM	Gray-Level Run Length Matrix
GLDM	Gray-Level Dependence Matrix
ICC	Intraclass Correlation Coefficient
LASSO	Least Absolute Shrinkage and Selection Operator
LR	Logistic Regression
MLP	Multilayer Perceptron
SVM	Support Vector Machine
XGBoost	Extreme Gradient Boosting
ROC	Receiver Operating Characteristic
AUC	Area Under the Curve
CI	Confidence Interval
TCGA	The Cancer Genome Atlas
RHWU	Renmin Hospital of Wuhan University
CMP	Corticomedullary Phase
